# Blockade of IL-33R/ST2 Signaling Attenuates *Toxoplasma gondii* Ileitis Depending on IL-22 Expression

**DOI:** 10.3389/fimmu.2019.00702

**Published:** 2019-04-18

**Authors:** Bernhard Ryffel, Feng Huang, Pauline Robinet, Corine Panek, Isabelle Couillin, François Erard, Julie Piotet, Marc Le Bert, Claire Mackowiak, Marbel Torres Arias, Isabelle Dimier-Poisson, Song Guo Zheng

**Affiliations:** ^1^Department of Clinical Immunology, Sun Yat-sen University Third Affiliated Hospital, Guangzhou, China; ^2^INEM UMR 7355 CNRS and University of Orleans, Orléans, France; ^3^Immunology and Virology Laboratory, Nanoscience and Nanotechnology Center, Universidad de las Fuerzas Armadas, ESPE, Sangolquí, Ecuador; ^4^UMR 1282 Infectiologie Animale et Santé Publique, Université de Tours -INRA, Tours, France; ^5^Department of Internal Medicine, Ohio State College of Medicine, Columbus, OH, United States

**Keywords:** *Toxoplasma gondii*, IL-33/ST2 receptor, neutralizing antibody, IL-22, parasite-induced ileitis, innate immunity

## Abstract

Oral *T. gondii* infection (30 cysts of 76K strain) induces acute lethal ileitis in sensitive C57BL/6 (B6) mice with increased expression of IL-33 and its receptor ST2 in the ileum. Here we show that IL-33 is involved in ileitis, since absence of IL-33R/ST2 attenuated neutrophilic inflammation and Th1 cytokines upon *T. gondii* infection with enhanced survival. Blockade of ST2 by neutralizing ST2 antibody in B6 mice conferred partial protection, while rmIL-33 aggravated ileitis. Since IL-22 expression further increased in absence of ST2, we blocked IL-22 by neutralizing antibody, which abrogated protection from acute ileitis in ST2 deficient mice. In conclusion, severe lethal ileitis induced by oral *T. gondii* infection is attenuated by blockade of ST2 signaling and may be mediated in part by endogenous IL-22.

## Introduction

*Toxoplasma gondii* is an opportunistic parasite with a worldwide distribution triggering an innate immune response. This response characterized by a rapid recruitment of neutrophils following the entry of infectious tachyzoites from the lumen into the intestinal mucosa eliciting a strong inflammatory Th1 response associated with the production of IFNγ, IL-12 and TNF-α. The parasite activates dendritic cells and macrophages to produce IL-12 leading to IFNγ expression (1). IL-17A is involved in neutrophil recruitment following infection, important for host defense and enhances a Th17 response via IL-17RA signaling (2). We found that IL-17RA deficient mice and B6 mice treated with neutralizing IL-17A antibody are more resistant to *T. gondii* induced acute ileitis as compared to infected B6 mice, suggesting that IL-17A contributes to the pathology of *T. gondii* inflammation (3).

IL-33, previously known as IL1F11 or nuclear factor from high endothelial venules (4), is a member of the IL-1 cytokine family (1, 5). IL-33 in the nucleus is associated with chromatin, but the role of nuclear IL-33 is not yet clarified (6). Upon cell stress or death, biologically active IL-33 is released and truncated by proteolytic cleavage (7). IL-33 may have a dual role in different inflammatory conditions, depending on the specific immune mechanisms underlying disease pathogenesis (5). IL-33R/ST2 is a stable cell marker on Th2 cells and innate immune cells (8). IL-33 induces the production of high amounts of the Th2 cytokines IL-5 and IL-13 by type-2 innate lymphoid cells in the intestine and the lung. The IL-33-IL-33R/ST2 axis is involved in inflammatory bowel diseases (IBD) (9, 10) and has a regulatory role in experimental mouse models of IBD. IL-33 controls intestinal permeability and negatively regulates wound healing in the colon (11), further supporting the notion that the IL-33-IL-33R/ST2 axis may represent an effective therapeutic target in IBD.

We showed before that oral infection with cysts of *T. gondii* (76K strain) caused upregulation of IL-1β and IL-17A in the ileum with acute lethal ileitis in sensitive B6 mice. Furthermore, both IL-1β and IL-17A are involved in acute inflammation of the proximal intestine caused by tachyzoites invasion of the mucosa (3, 12), while IL-22 confers protection (13).

Here we report a critical role of IL-33R/ST2 upon *T. gondii* infection (76K strain). Both ST2 and IL-33 are upregulated in the intestine and IL-33R/ST2 deficient mice have attenuated ileitis with increased IL-22 expression. Furthermore, the blockade of IL-22 by antibody neutralization reversed the protective effect found in IL-33R/ST2 deficient mice. Therefore, the data suggest that protection may be mediated by upregulation of the protective cytokine IL-22.

## Results

### Increased IL-33 Expression in *T. gondii* Induced Acute Ileitis

**Figure 1:**
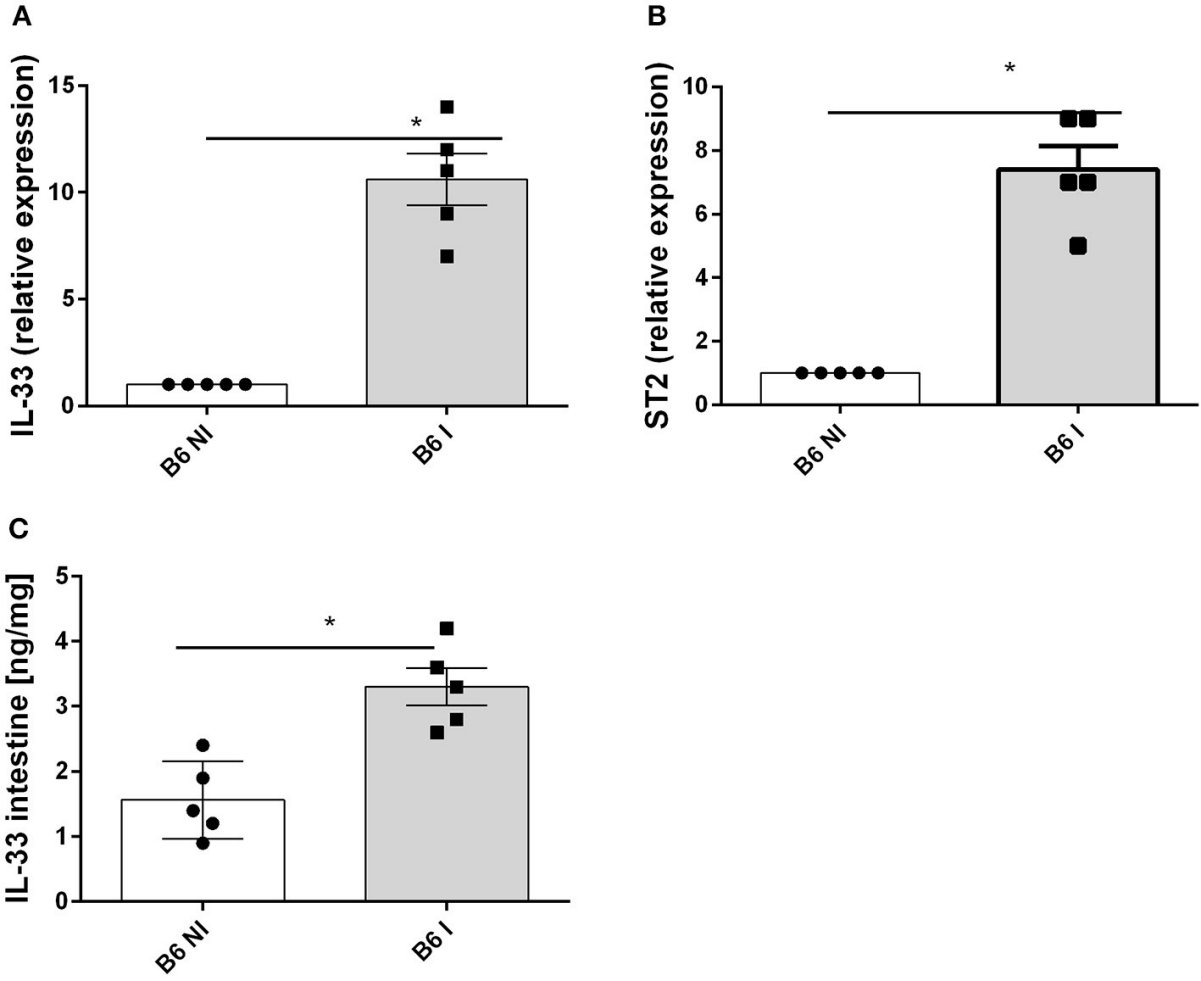
*T. gondii* infection induces IL-33 and ST2 expression in the ileum. B6 mice were infected by gavage with 35 cysts of *T.gondii* (76K strain) and transcripts were measured by q-PCR in the homogenate of the proximal ileum (2 cm of jejunum) for IL−33 **(A)** and ST2 mRNA **(B)** and IL-33 protein by ELISA in intestinal homogenate **(C)** at day 7 post-infection. Values are representative of two independent experiments expressed as mean ± SEM. ^*^, ^**^, and ^***^ refer to *P* < 0.05, *P* < 0.01, and *P* < 0.001, respectively.

Oral infection with 30 cysts of *T. gondii* (76K strain) causes a rapid upregulation of IL-33 and IL-33R/ST2 gene expression in the proximal ileum in C57BL/6 mice on day 7 (Figures 1A,B). Furthermore, IL-33 protein increases in the ileal mucosa (Figure 1C). To determine the source of IL-33 we performed immunostaining and found that IL-33 is expressed in the intestinal epithelium as well as myeloid and fibroblast like cells in the lamina propria as reported before (14). Therefore, we questioned whether IL-33 contributes to the inflammatory response in *T. gondii* infected mice.

### Diminished Intestinal Cytokine Production in the Absence of IL-33R/ST2

**Figure 2:**
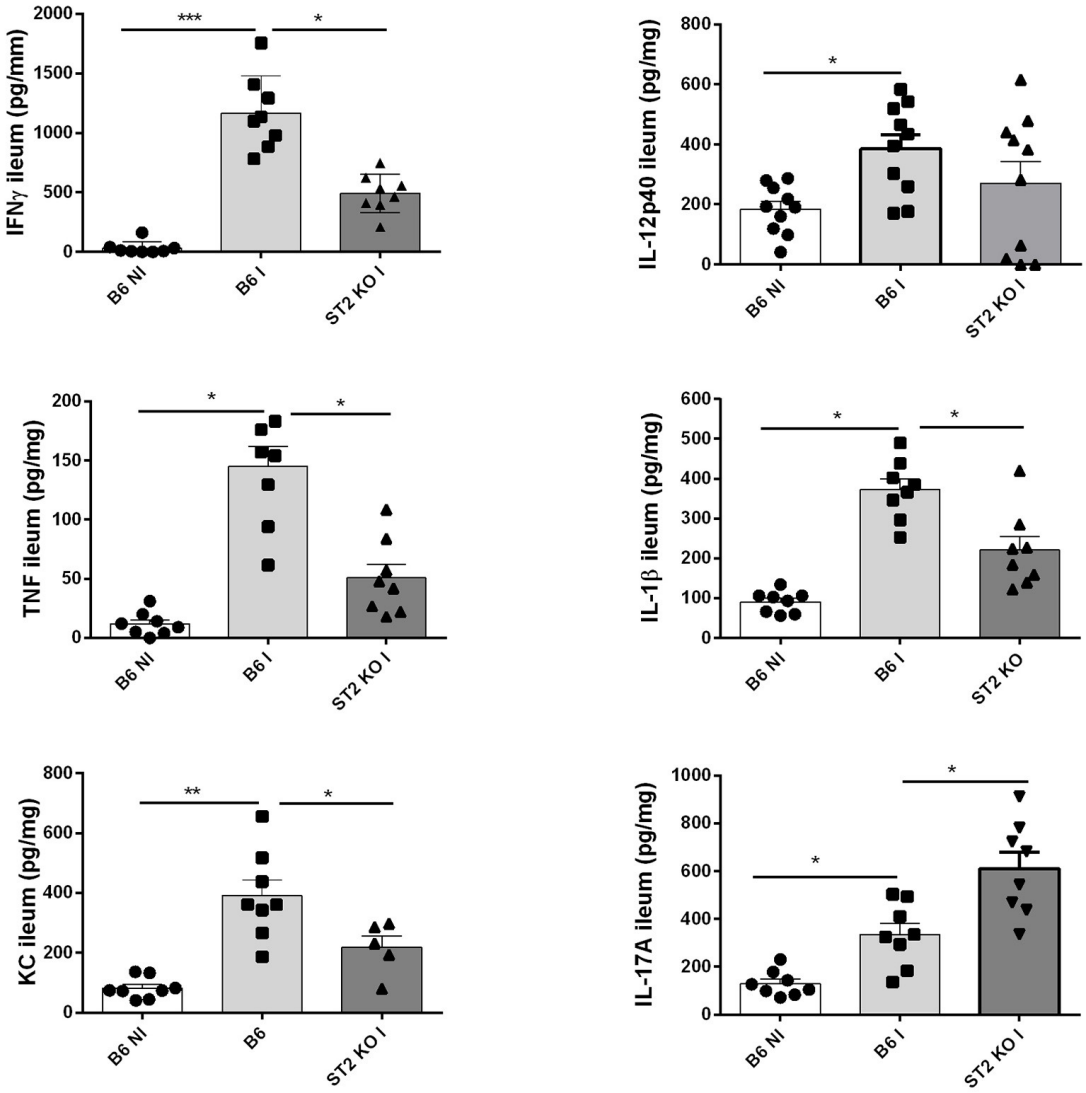
Reduced cytokine and chemokine production in the absence of IL-33R/ST2. Levels of IFNγ, TNF, IL-12p40, IL-1β, CXCL1/KC, and IL-17A were determined by ELISA in homogenates of the proximal ileum at day 7 post-infection. Values are representative of two independent experiments expressed as mean ± SEM.

Since IL-33 induces a proinflammatory response, we determined the cytokine profile in the mucosa of the ileum upon oral *T. gondii* infection, which in B6 mice has a Th1 signature. We confirm increased production of Th1 cytokines IFNγ, TNF, IL-12, IL-23, and IL-1β in the proximal ileum in B6 mice upon infection, while absence of IL-33R/ST2 attenuated the Th1 cytokine response (Figure 2). Moreover, an enhanced Th17 response with elevated IL-17A and IL-22 expression has been reported upon *T. gondii* infection with diminished ileitis in IL-17RA and IL-22 deficient mice (3, 13). Here we find augmented IL-17A tissue levels in infected B6 mice, which further increased in IL-33R/ST2 deficient mice (Figure 2). In conclusion, infection with *T. gondii* induces proinflammatory cytokine and chemokine responses, which are reduced in absence of IL-33R/ST2 signaling. Therefore, we asked whether blockade of this pathway would attenuate acute ileitis.

### *T. gondii* Induced Ileitis Is Attenuated in IL-33R/ST2 Deficient Mice

**Figure 3:**
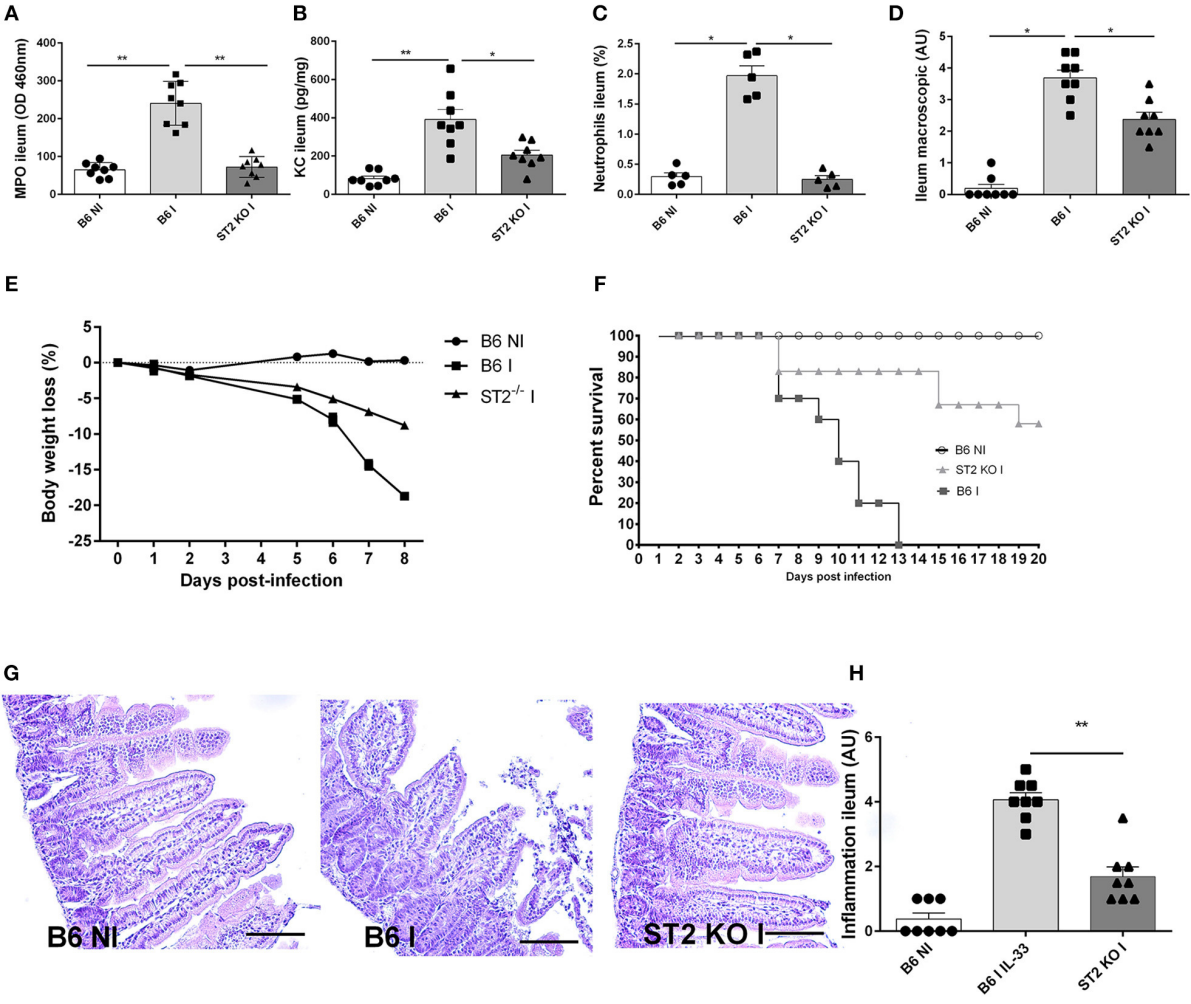
*T. gondii* induced ileitis is attenuated in IL-33R/ST2 deficient mice. Reduced MPO activity, KC and neutrophils recruitment in the ileum of IL-33R KO mice **(A–C)**, macroscopic signs of colon inflammation **(D)** and body weight loss **(E)** with enhanced survival of IL-33R/ST2 mice **(F)** associated with reduced severity of microscopic inflammation in the proximal ileum in the absence of ST2 **(G)** and semi-quantitative score of inflammation **(H)**. Analysis at day 7 post-infection. Values are representative of two independent experiments expressed as mean ± SEM.

Using IL-33R/ST2 deficient mice, we observe a reduced severity of *T. gondii* induced ileitis. Clinical signs of disease with loss of body weight and macroscopic inflammatory alterations of the ileum are diminished in IL-33R/ST2 deficient mice (Figures 3D,E). Increased CXCL1/KC levels are associated with enhanced MPO activity and neutrophil recruitment in the mucosa of the proximal ileum, which are significantly lower in the absence of IL-33R/ST2 (Figures 3A–C) and associated with enhanced survival as compared to B6 mice (Figure 3F). Microscopic analysis reveals reduced inflammation at day 7 of *T. gondii* infected IL-33R/ST2 deficient mice (Figures 3G,H). While infected B6 mice displayed severe acute inflammatory changes in the ileum, the severity of inflammation was attenuated in IL-33R/ST2 deficient mice (Figures 3G,H). Therefore, IL-33R/ST2 signaling mediates severe acute inflammation in the proximal ileum, which is fatal, but significantly reduced in the absence of IL-33R/ST2 signaling.

### IL-33R/ST2 Antibody Blockade Dampens, While Exogenous IL-33 Enhances *T. gondii* Induced Ileitis

**Figure 4:**
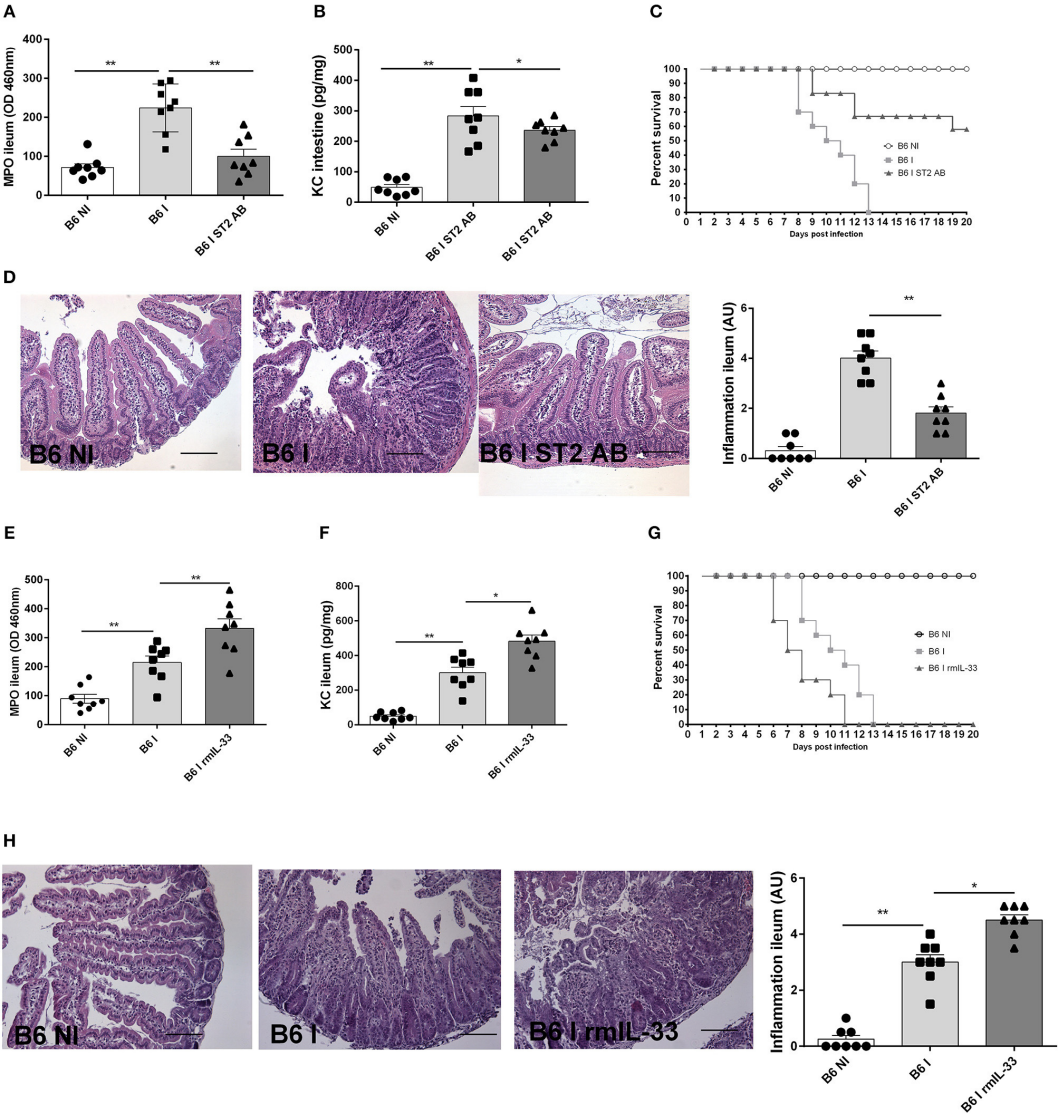
Neutralization of IL-33 attenuates, while rmIL-33 aggravates ileitis in BL6 mice. Antibody IL-33R/ST2 neutralization reduces MPO activity **(A)** and CXCL1/KC **(B)**, and enhances survival **(C)** associated with attenuated microscopic inflammation **(D)**. By contrast, rmIL-33 augments MPO activity, CXCL1/KC, survival and severity of Inflammation **(E–H)**. Analysis at day 7 post-infection. Values are representative of two independent experiments.

To ascertain that the protection observed is not a particularity of the IL-33R/ST2 deficient mice, we used neutralizing IL-33R/ST2 antibody in infected B6 mice and confirm a protective effect as observed in IL-33R/ST2 deficient mice. Neutrophil recruitment as measured by MPO activity and the chemokine CXCL1/KC are reduced in ileum comparable to that found in IL-33R/ST2 deficient mice (Figures 4A,B). Survival is significantly prolonged and severity acute ileitis reduced as shown for *T. gondii* infected IL-33R/ST2 deficient mice (Figures 4C,D).

To further confirm a critical role of IL-33, we investigated whether exogenous IL-33 enhances *T. gondii* induced inflammation. The injection of rmIL-33 (0.5 μg daily by i.p. route) in infected B6 mice augmented the inflammatory response with increased MPO activity and KC expression in the ileum and reduced survival due to enhanced severity of the ileitis confirmed by microscopic analysis (Figures 4E-H). Therefore, the data strongly suggests that IL-33 signaling via IL-33R/ST2 is critical for the inflammatory response in *T. gondii* induced ileitis.

### Enhanced IL-22 Expression Contributes to Attenuate Ileitis in IL-33R/ST2 Deficient Mice

**Figure 5:**
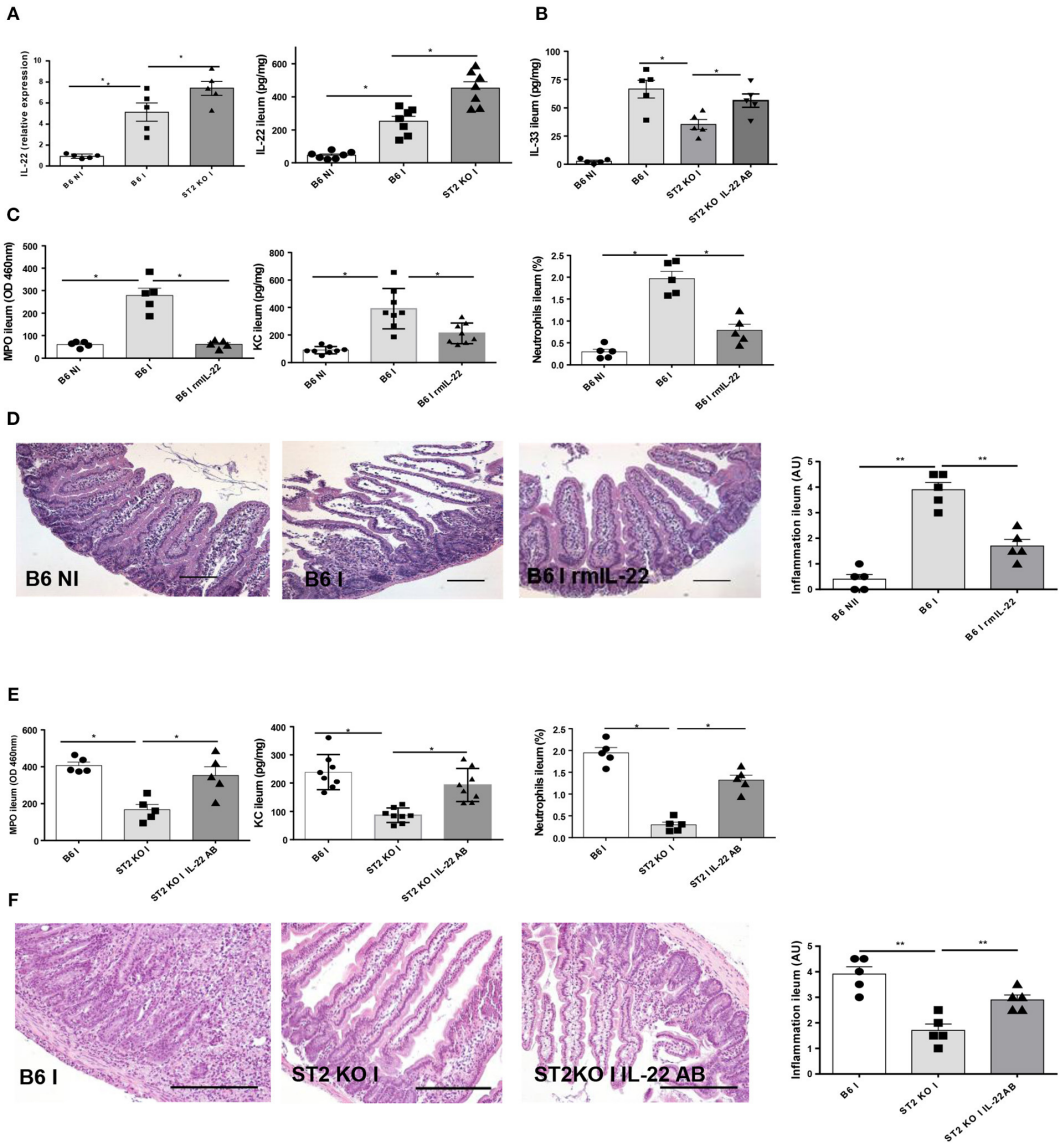
IL-22 confers protection in IL-33R/ST2 deficient mice upon *T. gondii* infection Infection by *T. gondii* infection induces enhanced IL22 mRNA and protein expression in ST2 deficient mice **(A, B)** and increased IL-33 expression **(B)**. Administration of rmIL-22 reduces MPO activity, CXCL1/KC levels, neutrophil recruitment **(C)** and severity of ileitis in B6 mice **(D)**. By contrast, IL-22 antibody blockade in IL-33R/ST2 deficient mice increased MPO activity, CXCL1/KC levels and neutrophil recruitment **(E)** and enhanced severity of ileitis **(F)**. Analysis at day 7 post-infection. Values are representative of two independent experiments.

IL-22 has a protective effect as reported before in *T. gondii* induced acute ileitis (3, 13). We revisited IL-22 expression and found increased IL-22 expression in the parasitized ileum of IL-33R/ST2 deficient mice (Figure 5A). IL-33 tissue levels are decreased in IL-33R/ST2 deficient mice, but IL-22 antibody neutralization augmented local IL-33 levels (Figure 5B).

Therefore, we asked whether exogenous rmIL-22 contributes to the protective effect. First, we injected rmIL-22 in infected B6 mice, and found a significant reduction of MPO activity, KC and neutrophil recruitment (Figure 5C) and attenuated severity of ileitis (Figure 5D) with enhanced survival in B6 mice (data not show) consistent with our previous results (13).

In view of increased IL-22 expression and a protective effect of exogenous IL-22, we hypothesized that IL-22 blockade by neutralizing antibody may reduce protection from acute ileitis in IL-33R/ST2 deficient mice. Indeed, we showed that IL-22 antibody administration (100μg i.p. injection) largely abrogates protection from acute neutrophil recruitment and tissue inflammation in the infected ileum (Figures 5E, F). Therefore, rmIL-22 attenuates *T. gondii* induced inflammation. Furthermore, endogenous IL-22 contributes to the protection observed in the absence of IL-33R/ST2 signaling. The data suggests that IL-33 may suppress the protective action of endogenous IL-22 in *T. gondii* infection.

## Discussion

Here we used oral *T. gondii* infection (76K strain) induced severe ileitis in mice which may serve as a model of IBD (15). We reported that IL-33R/ST2 upon *T. gondii* infection induced ST2, IL-33 and IL-22 expression in the intestine. We discovered that IL-33R/ST2 deficient mice have attenuated ileitis associated with increased IL-22 expression. Since IL-22 antibody neutralization reversed the protective effect in IL-33R/ST2 deficient mice, we conclude that protection may be related to increased expression of the cytokine IL-22, known for its protective function (13).

IL-33 has an important regulatory roles in IBD as reviewed before (10) and we demonstrated enhanced healing in experimental IBD models (11). However, the role of IL-33 or IL-33R/ST2 upon *T. gondii* induced ileitis is unknown. Previous work demonstrated that neuroinflammation induced by *T. gondii* is IL-33-dependent, since IL-33R/ST2 deficient mice have increased parasite growth and severe cerebral toxoplasmosis, but the ileitis was not investigated (16). The transcription factor trefoil 2 (TFF2) has been shown to regulate IL-33 expression and Th2 differentiation (17), while in absence of TFF2, a Th1 response prevailed. Infected TFF2 deficient mice displayed low parasite replication and reduced intestinal inflammation upon *T. gondii* infection, whereas B6 mice experienced uncontrolled inflammation with lethal outcome (18).

The resistance to develop ileitis observed in IL-33R/ST2 deficient mice is replicated in infected B6 mice administered ST2 neutralizing antibody. Similar data of attenuated inflammation have been reported for other IL-33 dependent inflammatory conditions (1, 5). Further, rmIL-33 enhanced acute ileitis in B6 mice. Therefore, IL-33 appears to be critical for the control of *T. gondii* induced ileitis and we asked whether other inflammatory cytokine are involved.

We reported before that IL-22 has a protective effect, since IL-22 deficient mice develop acute ileitis (13) and showed here that IL-22 administration reduced epithelial barrier injury and inflammation. By contrast, IL-17 another Th17 cytokine enhances *T. gondii* induced ileal inflammation, since IL-17RA deficient mice were protected (3). IL-10, another member of the broader IL-22/IL-10 cytokine family, plays a critical role in IBD as demonstrated by the spontaneous development of IBD in IL-10 deficient mice (19, 20). Interestingly, *T. gondii* infection in a model of IL-10 deficient intraepithelial lymphocyte transfer or NKT cell deficient (Jalpha281(-/-) mice had reduced ileitis with IL-10 expression (21, 22). Whether IL-10-dependent mechanisms contribute to the protective IL-33/ST2/IL-22 pathway has not been reported so far. Here we focused on the contribution of IL-22 in IBD, but investigations on the role of IL-10 in the protective IL-33/ST2/IL-22 axis deserves further investigations in the future.

The finding that IL-33R/ST2 signaling suppresses IL-22 is novel, but a recent study revealed IL-33 regulates IL-17A and IL-22 in fungal infection (23). IL-22 neutralizing antibody administration converted resistance to ileitis of IL-33R/ST2 deficient mice to an inflammatory Th1 phenotype, which may be due to enhanced prostaglandin E2 production (23). A role of increased expression of amphiregulin by IL-33 has been shown to contribute to control experimental colitis (14), which merits further investigations. Thus, alternative pathways may be considered such as the activation of the NLPR3 inflammasome complex (24), which is supported by reduced *T.gondii* induced ileitis in IL-1R1 deficient mice (12). The possibility that NLRP3/caspase-1 activation in *T.gondii* infection contributes to the down-modulation of IL-33 and the Th2 response should be considered as reported in allergic lung inflammation (25). Finally, in view of the critical role of neutrophils in *T.gondii* induced inflammation the polarization of neutrophils to express IL-17A may be of interest as previously shown in an ischemia reperfusion repair model (26).

In summary, IL-33R/ST2 signaling enhances *T. gondii* parasite-induced inflammation and IL-22 has an important protective effect (13). Resistance in absence of IL-33R/ST2 appears to be mediated by endogenous IL-22. Therefore, the beneficial effect of IL-22 administration on toxoplasma-induced ileitis may be relevant for human IBD of different origins.

## Materials and Methods

### Mice

*IL-33R/ST2* T1/ST2-deficient mice (27) were back-crossed 8 times on C57BL/6J genetic background and bred with wild-type littermates in our animal facility at the Transgenose Institute (CNRS, TAAM, Orleans, France). All animal experimental protocols complied with the French ethical and animal experiments regulations (see Charte Nationale, Code Rural R 214-122, 214-124 and European Union Directive 86/609/EEC) and were approved by the “Ethics Committee for Animal Experimentation of CNRS Campus Orleans” (CCO), registered (N°3) by the French National Committee of Ethical Reflexion for Animal Experimentation (CLE CCO 2012-042).

### Inoculation of *T. gondii* Cysts and Administration of IL-33, IL22, IL-33R, and IL-22 Neutralizing Antibodies

C57BL/6 (B6) and IL-33R/ST2 deficient mice were inoculated by gavage with 30 cysts of the 76K strain obtained as described before (13). Groups of 5 to 8 female mice of 8–12 weeks were used and the studies were repeated twice. Additional groups of mice were injected daily intraperitoneally with 0.5 μg of rm IL-33 (aa 109–266) or 5 μg rmIL-22 daily R&D system). Neutralizing rmIL-22 (R&D system) antibody and IL-33R/ST2 (gift from Dr. Dirk Smith, Amgen) antibody were injected at 50 μg per mouse or isotype control (rat IgG1, R&D system) on days 1, 3, and 5 after oral infection. The mice were analyzed at day 7 for neutrophil recruitment in the ileum and morphological alterations of the proximal ileum and additional groups were used for survival.

### RNA Extraction and PCR in Ileum

Ileum from control and infected B6 mice was collected, snap-freezed in liquid nitrogen and kept at −80°C. Total RNA were isolated from 100 mg of intestinal tissue homogenized with 1 mL of TRI Reagent^®^ (Sigma) using TRIzol/Chloroform extraction as described (13). RNA was then precipitated in isopropanol, washed with 75% ethanol and resuspended in RNase-free water. Reverse transcription was performed on 1 μg of RNA using GoScript Reverse transcription system (Promega). Quantitative real-time PCR were realized on cDNA obtained using primers for Il22, Il33, and St2 (Qiagen), GoTaq® qPCR-Master Mix (Promega) and detected on a Stratagene Mx3005P (Agilent technologies). At the end of the PCR amplification, a DNA melting curve analysis was carried out to confirm the presence of a single amplicon. *Gapdh* expression was used for normalization of transcript levels. Relative mRNA levels were determined using (2^−ΔΔ*Ct*^) method, determined by comparing (*i*) the PCR cycle thresholds (Ct) for the gene of interest and *Gapdh* (ΔCt) and (*ii*) ΔCt values for treated and control groups (ΔΔCt).

### Cytokine Measurement

Cytokine production for IFNγ, CXCL1/KC, IL-1β; IL-12p40, TNF-α, IL-17A, and IL-22 was evaluated in proximal ileum homogenate using commercial ELISA kits according to the manufacturer's instructions. Concentrations were normalized with organ weight and expressed in quantity per mg of tissue.

### Myeloperoxidase Activity (MPO) in Ileum

MPO activity was evaluated in tissues of the small intestines as described. In brief, the right heart ventricle was perfused with saline to flush the vascular content and ileum was frozen at −20°C until use. Ileum was homogenized in PBS by Ultra Turrax, centrifuged and the supernatant was discarded. The pellets were resuspended in 1 ml PBS containing 0.5% hexadecyltrimethyl ammonium bromide (HTAB) and 5 mM ethylene-diamine tetra-acetic acid (EDTA). Following centrifugation, 150 μl of supernatants were placed in test tubes with 200 μl PBS-HTAB-EDTA, 1 ml Hanks' balanced salt solution (HBSS), 100 μl of o-dianisidine dihydrochloride (1.25 mg.ml^−1^), and 100 μl H_2_O_2_ 0.05%. After 15 min of incubation at 37°C in an agitator, the reaction was stopped with 100 μl NaN_3_ 1%. The MPO activity was determined as absorbance at 460 nm against medium.

### Isolation of Lamina Propria Mononuclear Cells and Flow Cytometry

The small bowel was flushed with PBS and opened longitudinally, cut into 1 cm pieces and incubated in PBS/EDTA 3 mM during 20 min at 37°C under magnetic agitation. Pieces were then cut into 1 mm pieces and incubated in RPMI containing 0.5 mg/mL type IV collagenase (Life technologies), 1 ng/mL DNase (DN25, Sigma), 5% FCS (Perbio) and incubated 15 min at 37°C under magnetic agitation. Tissue debris and cell aggregates were removed by passage several times over a 10 mL syringe. Cells were filtered on 70 μm cells strainers and centrifuged 7 min at 1,700 rpm. Cells pellets were resuspended in 40% Percoll faction, overlayed on the top of a 80% Percoll fraction and centrifuged 20 min at 3,000 rpm without brake. LPMCs are collected in a white ring at the interphase of the two different percoll solutions and washed by RPMI 1640. Cells were then suspended in RPMI 1640 for experiments and 105 cells/mouse were stained by anti-CD11b PerCP Cy5.5 (Clone M1/70, BD Pharmingen) and anti-Ly6G PE-Cy7 (Clone RBL6-8C5, eBioscience) antibodies or by control isotypes in presence of Fc Block (anti-CD32/CD16) (Clone 24.G2, BD Pharmingen). FACS staining was assessed on a BD CANTO II cytometer and analyzed with FlowJo Software as described (13).

After 15 min of incubation at 37°C in an agitator, the reaction was stopped with 100 μl NaN_3_ 1%. The MPO activity was determined as absorbance at 460 nm against medium.

### Macroscopic and Microscopic Investigations

Proximal jejunum was collected 7 days after the infection, macroscopically observed to identify major alterations, fixed in 4% buffered formaldehyde and processed under standard conditions. Tissue sections (3 μm) were stained with haematoxylin and eosin. The inflammatory cell infiltrate with epithelial lesion was assessed by a semi-quantitative score from 0 to 5 (with increasing extent) by two independent, blinded experts (BR and PR) as described before (13).

### Statistical Analysis

Data were analyzed using Prism version 5 (Graphpad Software, San Diego, CA). The non-parametric Kruskal-Wallis test with Dunn's multiple comparison test or the parametric one-way ANOVA test with multiple Bonferroni's comparison test were used. Data were considered significant when *p* < 0.05 (^*^), 0.01 (^**^), 0.001 (^***^), or 0.0001 (^****^).

## Author Contributions

ID-P, SZ, FH, and BR conceived and designed the experiments. PR, CP, JP, MT, CM, and ML performed the experiments. PR, ID-P, CM, and BR analyzed the data. FE, IC, ML, and CM scientific advice. BR, PR, SZ, FE, and ID-P wrote the paper.

### Conflict of Interest Statement

The authors declare that the research was conducted in the absence of any commercial or financial relationships that could be construed as a potential conflict of interest.
