# Corrigendum: Blockade of IL-33R/ST2 Signaling Attenuates *Toxoplasma gondii* Ileitis Depending on IL-22 Expression

**DOI:** 10.3389/fimmu.2020.592865

**Published:** 2020-10-28

**Authors:** Bernhard Ryffel, Feng Huang, Pauline Robinet, Corine Panek, Isabelle Couillin, François Erard, Julie Piotet, Marc Le Bert, Claire Mackowiak, Marbel Torres Arias, Isabelle Dimier-Poisson, Song Guo Zheng

**Affiliations:** ^1^Department of Clinical Immunology, Sun Yat-sen University Third Affiliated Hospital, Guangzhou, China; ^2^INEM UMR 7355 CNRS and University of Orleans, Orléans, France; ^3^Immunology and Virology Laboratory, Nanoscience and Nanotechnology Center, Universidad de las Fuerzas Armadas, ESPE, Sangolquí, Ecuador; ^4^UMR 1282 Infectiologie Animale et Santé Publique, Université de Tours -INRA, Tours, France; ^5^Department of Internal Medicine, Ohio State College of Medicine, Columbus, OH, United States

**Keywords:** *Toxoplasma gondii*, IL-33/ST2 receptor, neutralizing antibody, IL-22, parasite-induced ileitis, innate immunity

In the published article, there was an error in affiliation 2. Instead of “UMR 7355 Université-CNRS INEM, Orléans, France and IDM, University of Cape Town, South Africa”, it should be “INEM UMR 7355 CNRS and University of Orleans, Orléans, France.”

In addition, the wrong microscopic plates were inserted into Figures 5E and F. The corrected Figure 5 appears below.

**Figure 5:**
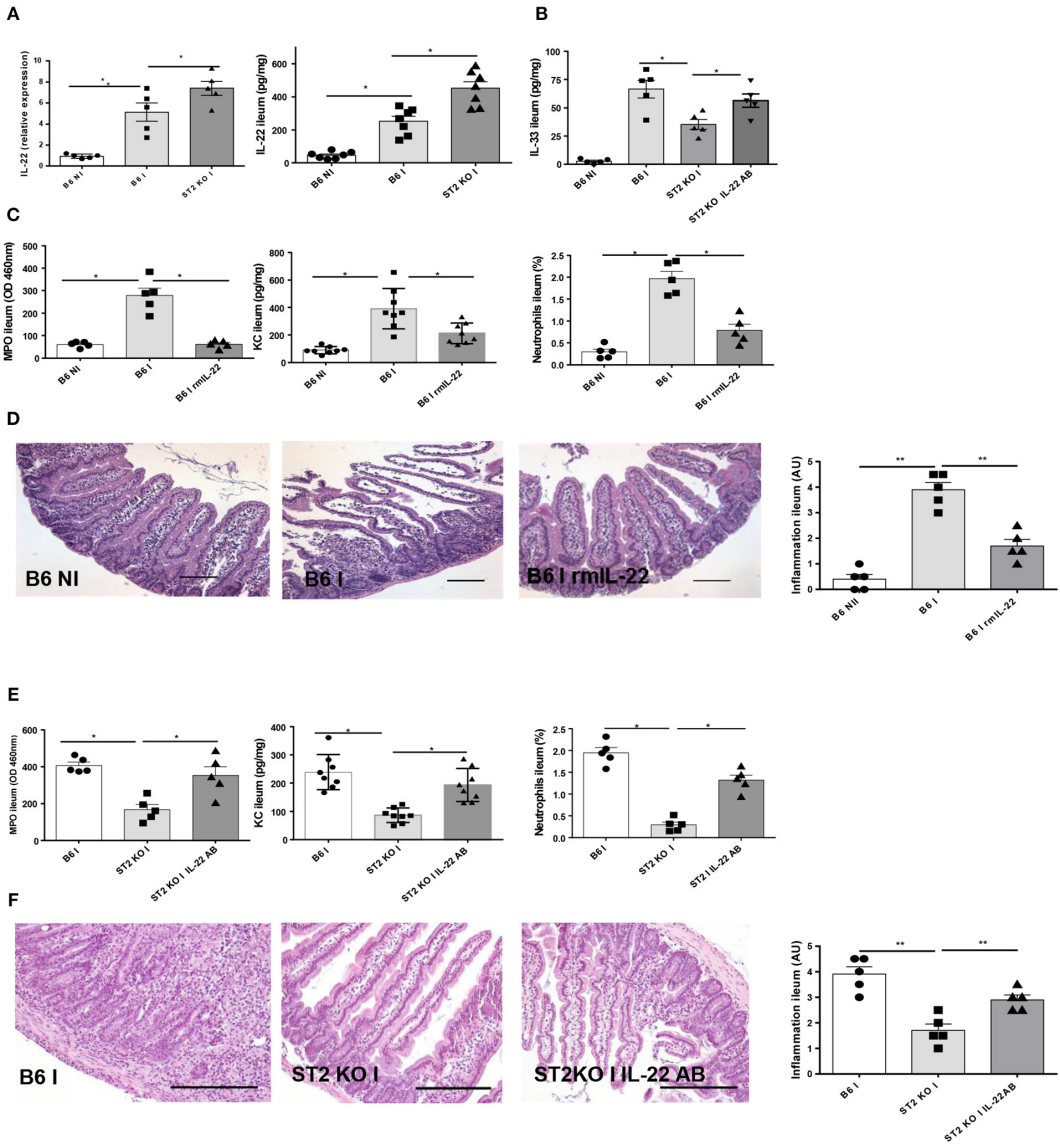
IL-22 confers protection in IL-33R/ST2 deficient mice upon *T. gondii* infection Infection by *T. gondii* infection induces enhanced IL22 mRNA and protein expression in ST2 deficient mice **(A,B)** and increased IL-33 expression **(B)**. Administration of rmIL-22 reduces MPO activity, CXCL1/KC levels, neutrophil recruitment **(C)** and severity of ileitis in B6 mice **(D)**. By contrast, IL-22 antibody blockade in IL-33R/ST2 deficient mice increased MPO activity, CXCL1/KC levels and neutrophil recruitment **(E)** and enhanced severity of ileitis **(F)**. Analysis at day 7 post-infection. Values are representative of two independent experiments.

The authors apologize for these errors and state that this does not change the scientific conclusions of the article in any way. The original article has been updated.

